# The effect of performance pressure and error-feedback on anxiety and performance in an interceptive task

**DOI:** 10.3389/fpsyg.2023.1182269

**Published:** 2023-05-12

**Authors:** David John Harris, Tom Arthur, Samuel James Vine, Harith Rusydin Abd Rahman, Jiayi Liu, Feng Han, Mark R. Wilson

**Affiliations:** School of Public Health and Sport Sciences, Exeter Medical School, University of Exeter, Exeter, United Kingdom

**Keywords:** attention, appraisal, choking, clutch, sport

## Abstract

**Introduction:**

Whilst the disruptive effects of anxiety on attention and performance have been well documented, the antecedents to anxiety in motivated performance scenarios are less well understood. We therefore sought to understand the cognitive appraisals that mediate the relationship between pressurised performance situations and the onset of anxiety.

**Methods:**

We tested the effects of performance pressure and error feedback on appraisals of the probability and cost of failure, the experience of anxiety, and subsequent impacts on visual attention, movement kinematics, and task performance during a virtual reality interception task.

**Results:**

A series of linear mixed effects models indicated that failure feedback and situational pressure influenced appraisals of the probability and cost of failure, which subsequently predicted the onset of anxious states. We did not, however, observe downstream effects on performance and attention.

**Discussion:**

The findings support the predictions of Attentional Control Theory Sport, that (i) momentary errors lead to negative appraisals of the probability of future failure; and (ii) that appraisals of both the cost and probability of future failure are important predictors of anxiety. The results contribute to a better understanding of the precursors to anxiety and the feedback loops that may maintain anxious states.

## 1. Introduction

The disruptive effects of anxious emotional states on the execution of sensorimotor skills has been well documented (Hill et al., 2010; Roberts et al., 2017; Payne et al., 2018; Cappuccio et al., 2019). Factors that increase the importance of performing well, known as *psychological pressure* (Baumeister, 1984), can induce a state of *anxiety*, which is comprised of cognitive worry and physiological arousal (Eysenck, 2013). Several mechanistic accounts of how anxiety subsequently impairs performance have been proposed (Eysenck et al., 2007; Masters and Maxwell, 2008; Wilson, 2008; Vine et al., 2016). These accounts have focused on the role of attention, which is believed to disrupt performance when directed towards irrelevant or threatening stimuli (distraction theories) (Lee and Grafton, 2015) or turned inwards to consciously control movements (self-monitoring theories) (Sullivan et al., 2022). Important questions remain, however, about when and how anxiety disrupts performance; in particular, why do athletes cope on one occasion Whilst exhibiting catastrophic performance breakdowns in another?

**Figure 1:**
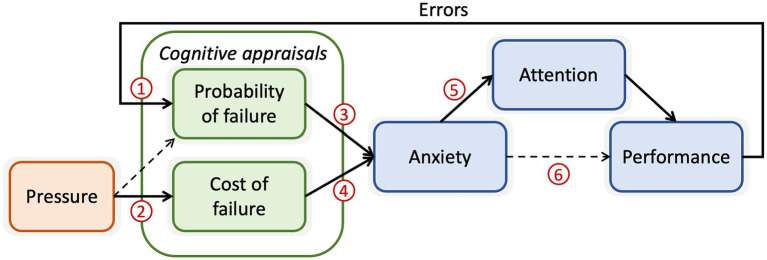
Schematic representation of the bi-directional pressure-performance relationship as outlined in ACTS. The blue boxes represent the indirect effect of anxiety on performance via attention, as outlined in ACT. Cognitive appraisals (green boxes) are thought to mediate the relationship between performance pressure and the state of anxiety. The arrow representing the feedback loop from performance to further cognitive appraisal of errors illustrates how momentary errors can influence appraisals of the probability of failure, which then further influence anxiety, in a continuous loop. The red numbers indicate relationships we sought to test in this study, which are described below.

In this work we adopted Attentional Control Theory: Sport (ACTS; Eysenck and Wilson, 2016) as a theoretical framework for understanding the precursors to choking. ACTS was originally developed for a sporting setting but has wider relevance for understanding disrupted motor control in the presence of anxiety. Attentional Control Theory (ACT; Eysenck et al., 2007), the predecessor of ACTS, proposed that anxiety causes increased attention to threat-related cues as a result of a disrupted balance between top-down and bottom-up attentional systems, which subsequently impairs performance efficiency. ACT, as a theory applied primarily to trait differences in anxiety, does not, however, explain the *origins* of anxious states. By contrast, ACTS (see Figure 1) proposes that the origins of anxiety are rooted in ongoing appraisals of the associated costs or consequences of undesirable outcomes and the probability of such outcomes. Specifically, ACTS draws on the work of Berenbaum et al. (2007) to use the concepts of ‘probability of failure’ (POF) and ‘cost of failure’ (COF) as mediators of the relationship between pressure and anxiety. These mediators explain the important fact that performance pressure (e.g., from social comparison or monetary reward) does not necessarily lead to anxiety. The onset of anxiety will instead depend on whether the performer thinks failure is likely (i.e., POF) and whether the consequences of failure are meaningful (i.e., COF). These appraisal processes bear a relationship with those proposed within the biopsychosocial model (BPSM) of challenge and threat (Blascovich, 2008). The BPSM proposes that individuals evaluate the demands of the task (demand evaluation) and whether they possess the necessary resources to cope effectively with these demands (resource evaluation), with the relative balance of the two determining whether the response to stress is threat or challenge. There is clear conceptual overlap between demand and resource evaluations and POF estimates, but the BPSM does not explicitly account for the predicted *cost* of failure, and hence ACTS provides an extended conceptualisation that could help to explain the onset of anxiety.

As well as outlining the cognitive appraisals that precipitate anxiety, ACTS also identifies the important role that feedback from momentary errors may play in generating anxiety; an issue that has received limited attention within sport psychology (despite interesting insights from qualitative research -see Nicholls et al., 2005). Within the ACTS framework, perceived POF increases as a function of the number of recent failure experiences. When combined with situations that are appraised as meaningful to the performer (high COF), anxiety may result. ACTS specifically predicts an interactive effect of POF and COF, whereby perceived probability of failure will have a greater impact when the cost of failure is also high. Attentional mechanisms could account for such an effect, as errors are more likely to be attended to and interpreted more negatively when anxiety is already high (i.e., increased attention to threat; Eysenck et al., 2007).

The overarching predictions of ACTS – that errors and pressure may interact to impair performance -have recently been supported in two studies of real-world sporting performance (Harris et al., 2019, 2021). Using large real-world datasets from American Football (seven seasons of the National football League; Harris et al., 2019) and elite Tennis (Grand Slam tournaments from 2016–2019; Harris et al., 2021), Harris and colleagues showed that situational pressure, prior errors, and their interaction all predicted performance errors in a subsequent play/point. In these studies, pressure was inferred using a cumulative scoring system based on game situations, such as a close score in the 4^th^ quarter for American Football, or a break point in tennis. As the game situation became more pressurised, errors (e.g., fumbling the football or double faulting in tennis) became more likely. Mistakes were also more likely on plays or points immediately after an error, which was further exacerbated when pressure was also high. Indeed, in the NFL, offensive errors were twice as likely to occur on a high pressure play following a preceding error, than following a low pressure, successful play (Harris et al., 2019).

These results from large real-world data sets (see also (Elmore and Urbaczewski, 2018; Evans and Crosby, 2021) in professional golf) provide compelling evidence that the type of pressure and error feedback effects predicted by ACTS are present in elite sport. Real-world data sets do not, however, allow us to interrogate the mechanisms responsible for these effects. In particular, we cannot know the cognitive appraisals that mediated the effects of pressure and prior errors on performance. Experimental studies to test these predictions are, therefore, a crucial next step. There is existing experimental support for feedback loops between anxiety and attentional biases in cognitive tasks. For instance, in a dot-probe task Liu et al. (2019) demonstrated a feedback loop between state anxiety and attentional bias, in which state anxiety directly increased attentional bias towards negative words and an experimentally-induced negative attentional bias increased state anxiety under stressful conditions. This relationship between attentional bias and state anxiety was found to be moderated by cognitive appraisals (see also Basanovic et al., 2020). The hypothesised relationships between POF, COF, anxiety, and errors outlined in Figure 1 are, however, yet to be tested and need to be studied in visuo*moto*r skills.

In the present work we used a virtual racquetball task to study the pressure-performance relationship as outlined in ACTS. We sought to test whether momentary fluctuations in anxiety were related to ongoing assessments of the probability and costs of failure, how performance errors influenced perceived POF and anxiety, and the downstream effects on attention and motor skill execution. To do this, we generated conditions of high and low pressure, and high and low performance failure feedback. We took frequent assessments of perceived POF, COF, and anxiety to measure momentary fluctuations that previous studies have largely ignored. Based on ACTS (Eysenck and Wilson, 2016) we generated the following hypotheses:

*H1*: More frequent feedback about performance errors will lead to higher POF ratings. This corresponds to testing relationship one in Figure 1.

*H2*: Higher pressure performance conditions will lead to higher COF ratings. This corresponds to testing relationship two in Figure 1.

*H3*: Higher pressure performance conditions and feedback about performance errors will lead to greater self-reported anxiety (overall effect of relationships one-four).

*H4*: Cognitive appraisals of POF and COF, and their interaction, will predict self-reported anxiety (relationships three and four in Figure 1).

*H5*: Momentary increases in self-reported anxiety will predict disruptions to visual attention (relationship five in Figure 1).

*H6*: Momentary increases in self-reported anxiety will predict disruptions to performance (both task outcomes and movement execution – relationship 6 in Figure 1).

*H7*: Errors on previous trials will predict further disruptions to performance (combined effect of relationships 1, 3, and 6 in Figure 1).

## 2. Methods

### 2.1. Design

This study adopted a repeated measures design with participants performing under two performance pressure conditions (high/low) and two failure feedback conditions (high probability of failure/low probability of failure).

### 2.2. Preregistration

Following completion of data collection, but before any formal analysis of the data, we pre-registered a set of hypotheses and analyses. The pre-registration document can be viewed here: https://osf.io/vebqs/. Any analyses that deviate from this plan are specified in the manuscript as exploratory.

### 2.3. Participants

Forty-three participants (ages 18–30 years, mean = 22.75 ± 2.3; 22 males, 21 females) were recruited from the population at a UK University to take part in the study. Participants were naïve to the aims of the experiment and reported no prior experience of playing VR-based racquet sports. They attended a single session of data collection for ~1.5 h. Three participants were removed totally from the dataset as they had difficulties with English language comprehension and did not understand the pressure manipulation, which was evident from repeated requests for clarification.

A series of power curves for the main tests of interest were generated using the simr package for *R* (Green and Mac Leod, 2016). Markov Chain Monte Carlo simulations were run to generate a simulation of observed power across a range of sample sizes, based on known variance in the dependent variables and an imputed minimum effect size of interest. For linear mixed effects models examining the effect of condition on POF and COF estimates, 43 participants were sufficient to detect small effects with more than 85% power for all the main analyses. Plots of the power curves, *R* code, and further details of the calculations can be found in the supplementary files (see: https://osf.io/vebqs/).

Ethical approval (Ref: 22–02-02-A-02) was provided by the University Ethics Committee before data collection and participants gave written informed consent prior to taking part. The study methods closely followed the approved procedures and the Declaration of Helsinki.

### 2.4. Task

Participants performed a racquetball task (previously reported in Arthur et al., 2021, 2022), in which they were required to hit oncoming balls back towards a target (the task code is available from the Open Science Framework: https://osf.io/ewnh9/). Interceptive skills, and the sensorimotor processes underpinning their control, have been well studied in the skill acquisition literature (Diaz et al., 2013a, 2013b; Mann et al., 2019; Arthur and Harris, 2021). Consequently, a range of relevant performance variables have been established which we adopted to understand skill breakdown under pressure.

Participants were placed in a virtual reality simulation of an indoor racquetball court, which spanned 15 m in length and width. Two targets were placed at the front of the court, one consisting of a series of concentric circles and another circle above it (height: 2 m) from which virtual balls were projected on each trial. The floor resembled that of a traditional squash court and participants were instructed to start behind the ‘short line’ (located 9 m behind front wall, 0.75 m from the midline). On each trial, the ball was projected towards the participant, who was instructed to hit it back towards the concentric circles target using a virtual racquet, operated by a hand controller. Virtual balls were 5.7 cm in diameter and had the visual appearance of a real-world tennis ball. The visible racquet in VR was 0.6 × 0.3 × 0.01 m, although its physical thickness was exaggerated by 20 cm for the detection of ball-to-racquet collisions.

The software for the VR task was developed using the gaming engine Unity 2019.2.12 (Unity technologies, CA) and C# programming language. The task was displayed through an HTC-Vive Pro Eye (HTC, Taiwan) head-mounted display, a high precision VR system which has proven valid for small-area movement research tasks (Niehorster et al., 2017). The Pro Eye headset is a 6-degrees of freedom, consumer-grade VR-system which allows a 360^o^ environment and 110^o^ field of view. Graphics were generated with an HP EliteDesk PC running Windows 10, with an Intel i7 processor and Titan V graphics card (NVIDIA Corp., Santa Clara, CA). Two ‘lighthouse’ base stations recorded movements of the headset and hand controller at 90 Hz. The headset has an inbuilt Tobii eye-tracking system, which uses binocular dark pupil tracking to monitor gaze at 120 Hz (spatial accuracy: 0.5–1.1°; latency: 10 ms, headset display resolution: 1440 × 1,600 pixels per eye). Eye position data was accessed in real-time using the SRanipal SDK.^1^

### 2.5. Procedures

**Figure 2:**
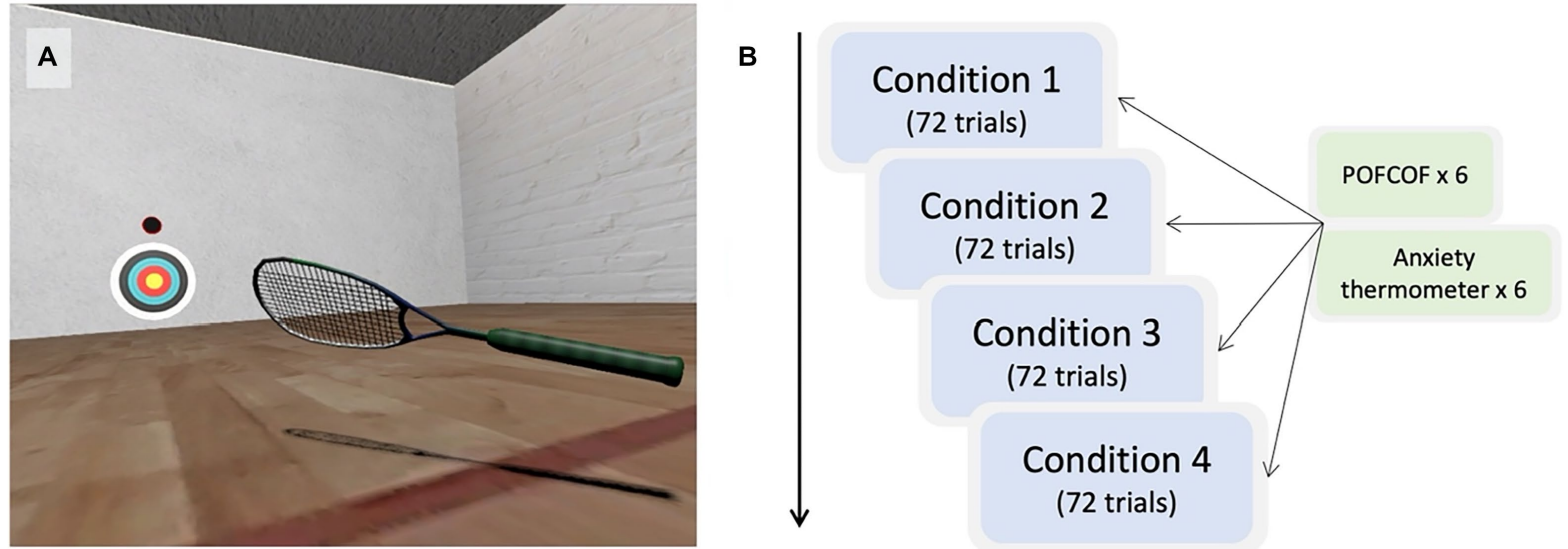
Image of the task environment **(A)** and schematic representation of the design of the study **(B)**. POFCOF = Probability of Failure and Cost of Failure measurement (see ‘Measures’).

On arrival at the laboratory, participants had the experimental tasks verbally explained to them and then provided written informed consent. Next, they were fitted with the VR headset and the inbuilt eye-tracking system was calibrated over five locations. It was also recalibrated after any displacement of the headset. During each trial, individuals were instructed to hit the oncoming ball back towards the centre of the projected target (see Figure 2, left). The release of each ball was signalled by three auditory tones. The ball passed exactly through the room’s midline, bouncing 3.5 m in front of the prescribed starting position. All participants were right-handed so started 0.75 m to the left of the midline so that all shots were forehand swings.

To generate additional challenge and variety in the task, the projected balls were of identical visual appearance but had two distinct elasticity profiles –one bounced like a normal tennis ball (65% elasticity) Whilst one had increased elasticity (85%). The two ball types followed the same pre-bounce trajectory and speed (vertical speed: −9 m/s at time of bounce), which was consistent with the effects of gravity (−9.8 m/s^2^). The ball made a bounce noise when it contacted the floor and then, crucially, it disappeared on contact with the racquet so that participants could be given bogus feedback about trial outcomes. Participants were told that the experimenter could still see where the ball went, but that they themselves could not. Participants completed five familiarisation trials of the interception task during which the ball was visible after interception, to convince them that the position of the ball was still being monitored. A short familiarization period was chosen as the task was relatively simple to execute and because we did not require participants to obtain any particular performance level beyond consistently intercepting the ball. Whilst returning the ball accurately to the target on the front wall was challenging, most participants are able to learn to intercept the ball (e.g., Arthur et al., 2021, 2022).

Participants completed four blocks of 72 trials, which corresponded to the high-and low-pressure conditions and the high-and low-performance failure feedback conditions. The conditions were conducted in a pre-determined order that was counterbalanced across participants. The pressure manipulation was conducted by presenting participants with one of two pre-recorded instruction videos (which can be viewed here: https://osf.io/vebqs/). In the low-pressure conditions, participants were instructed that the experimenters were using the data to compare to clinical populations with movement disorders and that they were simply providing baseline data for neurotypical individuals. In the high-pressure condition, participants were instead told that they needed to perform to the best of their ability because there was a monetary reward for the best performing individuals, and that low performing individuals would be interviewed for a video we were making on choking under pressure (i.e., inducing social evaluative threat). To create differences in performance failure feedback, the experimenter provided non-contingent feedback on each trial. ‘Hit’ or ‘miss’ was called after each trial, following a predetermined order that created conditions that were mostly successful (58% success –low probability of failure) or mostly unsuccessful (42% success –high probability of failure). These percentages were selected to create conditions that had a majority of either success or failure, but which were not implausibly different.^2^

POFCOF and anxiety thermometer self-reports (see 2.6.4 and 2.6.5 for explanation) were recorded after six pre-specified trials in each of the blocks of 72 trials (see Figure 2 for an illustration). These trials were carefully matched so that the probability of the two ball types was always the same (*p*(normal) = 0.67) and that the performance failure feedback to date always matched the overall ratio of hit to miss for that block. Half of these pre-specified trials were after hits and half after misses. After completing the four conditions, participants were thanked for taking part and debriefed about the anxiety manipulation.

### 2.6. Measures

#### 2.6.1. Performance

Task performance was assessed using the number of trials in which participants successfully made contact between the ball and racquet (interception rate), which was recorded within the VR environment. Whilst the bogus feedback given to participants referred to whether the ball hit the far target, this was not a valid performance variable because participants could not see the outcome and therefore could not adjust their performance from trial to trial. Whilst we were primarily interested in the processes underpinning performance efficiency, we used interception rate as an outcome measure of how accurately the ball was tracked (as in Arthur et al., 2021).

#### 2.6.2. Gaze variables

Interceptive performance is underpinned by a clear gaze strategy which involves predicting the future trajectory of the oncoming ball using anticipatory saccades (Land and McLeod, 2000). After initially tracking the flight of the ball, the eyes jump ahead and make a fixation at the predicted bounce location before the ball arrives. This fixation is known to be sensitive to both the early-flight trajectory of the ball and its predicted elasticity profile (Diaz et al., 2013a,b; Mann et al., 2019). After the ball bounces, observers attempt to track the ball onto the racquet through a combination of smooth pursuit and corrective (‘catch-up’) saccades. More accurate predictions of the bounce point enable better post-bounce tracking of the ball (Hayhoe et al., 2005). Anxiety is known to disrupt the functional coupling between perception and action and could therefore impair this visual tracking strategy (Nieuwenhuys and Oudejans, 2012; Ducrocq et al., 2016). In general, anxiety can lead to less goal-directed and more stimulus driven visual attention (Vine and Wilson, 2011), and as a result, metrics related to expert-like tracking may be sensitive to changes in anxiety. To test this hypothesis, we calculated the following two components of this predictive attentional control strategy: the bounce fixation location and duration.^3^

***Bounce fixation.*** A single fixation to a location a few degrees above the bounce point of an oncoming ball is a crucial part of intercepting a bouncing ball (Diaz et al., 2013a,b; Mann et al., 2019). The spatial position of this fixation (the gaze pitch angle) is sensitive to beliefs about likely ball trajectories (Diaz et al., 2013a,b) and a higher pitch angle is known to occur when there is greater uncertainty about the bounce of the ball (Diaz et al., 2013a,b; Arthur and Harris, 2021). The location of this fixation (referred to as the *gaze pitch angle* from here on) was calculated from the single unit gaze direction vector extracted from the inbuilt eye-tracking system (head-centred, egocentric coordinates). It was defined as the average gaze-head pitch angle (°) of the fixation that occurred at the time of, or immediately prior to, the ball bouncing. As previous studies examining the effect of anxiety on attention in visuomotor skills have also found that key task-related fixations can be disrupted (i.e., attenuated) under high pressure conditions (e.g., Causer et al., 2011; Vine and Wilson, 2011; Ducrocq et al., 2016), we also measured the duration of this bounce fixation. When intercepting a bouncing ball, expert-like gaze control is characterised by the eye arriving at the bounce point earlier (Mann et al., 2013), Whilst tracking fixations in other aiming tasks have been found to be shorter under anxiety (Causer et al., 2011). Consequently, we anticipated that anxiety could reduce the duration of this fixation.

#### 2.6.3. Swing kinematics

***Peak swing velocity***. To capture aspects of swing kinematics that may be influenced by anxiety, we calculated peak velocity of the hand, defined as the highest speed that the hand controller reached when moving towards the ball (expressed in metres/s). Higher peak velocities, which occur close to ball contact, are indicative of more proficient motor control (Reid et al., 2013) and therefore reductions in peak velocity may indicate disrupted motor control, as has been observed in many previous studies (Williams et al., 2002; Moore et al., 2012; Harris et al., 2023). Velocity of the hand controller was calculated from the square root of the sum of squared vector differentials during the foreswing phase (representing the forward phase of the hand movement before ball contact).

#### 2.6.4. Anxiety

State anxiety was measured at six timepoints during each condition using the anxiety thermometer (Houtman and Bakker, 1989). The anxiety thermometer is a 10 cm visual analogue scale on which participants were asked to rate their anxiety feelings at a particular moment, ranging from 0 (not anxious at all, the left end) to 10 (extremely anxious, the right end). It has been widely used in sporting tasks to perform a simple assessment of momentary anxiety (e.g., Pijpers et al., 2003). The thermometer had numerical anchors for ‘0’ and ‘10’ at the extremes of the scale.

#### 2.6.5. Perceived probability of failure and cost of failure (POFCOF)

Following Berenbaum et al. (2007), we measured perceived probability of failure (POF) and costs of failure (COF) using two 7-point Likert scales (see also (Payne, 2019)). The two scales were taken at six pre-determined points during each block of shots. Participants were asked to indicate how probable they think it is that they will miss the target with their next shot (POF; 0 = extremely unlikely; 6 = extremely likely), and how upset they would feel if they missed (COF; 0 = not at all upset; 6 = extremely upset).

### 2.7. Data processing

Positional data from the Vive hand controller, the headset, the direction of the eyes and the trajectory of the ball in the virtual environment were all recorded into csv files for each interception trial at a rate of 90 Hz. A single unit gaze direction vector of head-centred, egocentric coordinates (i.e., vertical and horizontal coordinates) was extracted from the Vive’s inbuilt eye-tracking system. This extracted gaze vector, and the ball’s head-centric position, were then plotted with respect to 2D direction in space, to provide relative ‘in-world’ angular orientation metrics. We refer to these orientations as *yaw* (rotation about a vertical axis that is in-line with gravity) and *pitch* (angular deviance from a plane originating at eye-height that is parallel to the floor plane). Using a bespoke MATLAB script, all trials were segmented from the moment of ball release until the time-point corresponding to ball contact frame. The contact point between the racquet and ball (referred to as ball contact frame) was identified from either an abrupt change in the ball trajectory or when the ball’s depth position was beyond that of the racquet for trials where the ball was missed.

Positional data from the hand controller and the position of the head were denoised using a dual-pass, zero-phase Butterworth filter (lowpass 10 Hz) (Franks et al., 1990). Gaze values were passed through a three-frame median filter, before being smoothed by a second-order, zero-lag Butterworth filter (Fooken and Spering, 2020). In line with recent recommendations (Cesqui et al., 2013, 2015), separate filter cut-off frequencies were applied for saccade identification (50 Hz) and analysis of positional tracking features (15 Hz). Trials with >20% missing data, or where eye-tracking was temporarily lost (>100 ms) were excluded. Angular velocities (degrees/s) and accelerations (degrees/s^2^) of gaze-in-world vectors were calculated from the distance between sequential samples of the filtered signal.

Fixations and saccades in the eye movement signal were calculated as follows. Saccades were identified from portions of data where gaze acceleration was more than five times its median absolute acceleration (Mann et al., 2019). To avoid erroneous detections arising from smooth pursuit or tracker-noise artefacts, gaze velocity was required to exceed 40°/s for five consecutive frames and had to be at least 20% greater than that of the ball to be counted as a saccade. Time periods preceded or followed by missing data were also excluded for being identified as a saccade. Onset and offset times of each saccade event were determined from the acceleration minima and maxima (Fooken and Spering, 2020). Separately, a spatial dispersion algorithm was used to extract gaze fixations (Krassanakis et al., 2014). A fixation event was operationalised as a portion of data where gaze remained within a 3° spatial dispersion threshold for a minimum duration of 100 milliseconds (Salvucci and Goldberg, 2000).

### 2.8. Statistical analysis

Data analysis was performed in RStudio v1.4.1106 (R Core Team, 2017). The dataset was first screened for outlying values on a per condition basis, with values more than 3 standard deviations from the mean (Tabachnick and Fidell, 1996) replaced with a Winsorized score. A series of linear mixed effects models (LMMs; fitted using restricted maximum likelihood in the lme4 package (Bates et al., 2014)) were then used to examine the stated research questions. The use of LMMs was critical for the focus on momentary fluctuations in anxiety. LMMs use multiple data points per participant, rather than averaging across blocks, and therefore allow changes in self-reported anxiety to be more accurately mapped onto changes in the dependent variables. For instance, if a participant’s anxiety increased or decreased over a block, the relationship between anxiety and the other variables could still be detected, making this analysis more sensitive to momentary fluctuations in anxiety. Model fit cheques were performed using the ‘performance’ package (Lüdecke et al., 2021) and can be accessed from the supplementary materials.^4^ When interpreting the results of the LMMs, we follow the effect size rules of thumb outlined in Acock (2014), which state that standardised beta effect sizes can be interpreted similarly to *r* (i.e., <0.2 is weak, 0.2–0.5 is moderate, and > 0.5 is strong). Otherwise stated, a standardised beta of 0.5 indicates that a one standard deviation change in the predictor variable equates to a half standard deviation change in the outcome variable.

## 3. Results

### 3.1. Effect of failure feedback and pressure instructions on POF, COF, and anxiety (H_1_, H_2_, H_3_)

**Figure 3:**
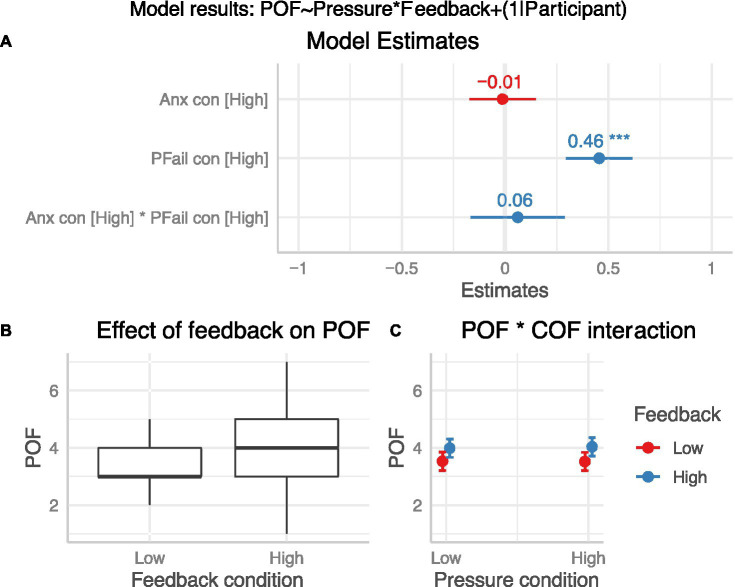
Effect of condition on POF (H_1_). Panel **(A)**: Plot of model β estimates with 95%CI error bars. Significant effects are indicated by an asterisk. Panel **(B)**: Boxplot of POF means between high and low failure feedback conditions. Panel **(C)**: Interaction effect (means and 95%CIs).

To address H_1_ and H_2_, we assessed whether the experimental conditions led to different POF and COF ratings. For POF ratings (see Figure 3), a linear mixed model with pressure and feedback conditions as fixed effects had a total *R*^2^ of 0.55 and marginal *R*^2^ of 0.03. The effect of failure feedback was statistically significant (*β* = 0.46, 95%CI [0.29, 0.62], *p* < 0.001; std. beta = 0.33), but there was no effect of pressure condition (*β* = −0.01, 95%CI [−0.17, 0.15], *p* = 0.89; std. beta = −0.01) and no interaction (*β* = 0.06, 95%CI [−0.17, 0.29], *p* = 0.60; std. beta = 0.04). This indicates that receiving feedback about task failure influenced appraisals of the probability of subsequent failure.

**Figure 4:**
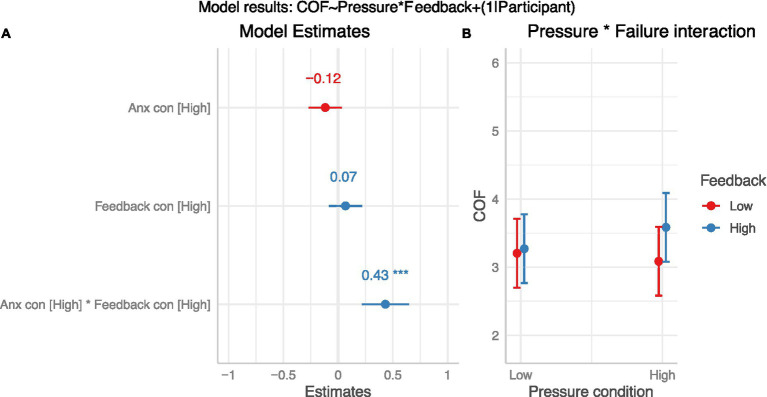
Effect of condition on COF (H_2_). Panel **(A)**: Plot of model β estimates with 95%CI error bars. Significant effects are indicated by an asterisk. Panel **(B)**: Interaction effect (means and 95%CIs).

For COF ratings (see Figure 4), the linear mixed model (with participant as a random factor) had a total *R*^2^ of 0.78 and marginal *R*^2^ of 0.01. The effect of both pressure (*β* = −0.12, 95%CI [−0.27, 0.03], *p* = 0.13; std. beta = −0.06) and failure feedback (*β* = 0.07, 95%CI [−0.09, 0.22], *p* = 0.39; std. beta = 0.04) were statistically non-significant. The interaction was, however, statistically significant (*β* = 0.43, 95%CI [0.22, 0.65], *p* < 0.001; std. beta = 0.23). Follow-up t-tests with Bonferroni-Holm correction showed that there was no difference in COF scores between low and high pressure for low failure feedback (*p* = 0.13), but there was a significant increase from low to high pressure when accompanied by high failure feedback (*p* < 0.001), suggesting that the effect of pressure was only present when the likelihood of failure was also high (see Figure 4B).

For self-reported anxiety, the linear mixed model (with participant as a random factor) had a total *R*^2^ of 0.71, but marginal *R*^2^ of less than 0.01. The effect of pressure condition (*β* = −0.04, 95%CI [−0.11, 0.03], *p* = 0.24; std. beta = −0.03), feedback condition (*β* = 0.05, 95%CI [−0.02, 0.13], *p* = 0.14; std. beta = 0.04), and their interaction (*β* = 0.07, 95%CI [−0.03, 0.17], *p* = 0.17; std. beta = 0.05) were all non-significant, showing that there was no difference in anxiety between high (*M* = 2.49, 95%CI [2.00, 2.97]) and low (*M* = 2.56, 95%CI [2.07, 3.05]) pressure, or between high (*M* = 2.66, 95%CI [2.17, 3.15]) and low failure feedback (*M* = 2.38, 95%CI [1.90, 2.87]).

### 3.2. Relationship between POFCOF and anxiety (H_4_)

**Figure 5:**
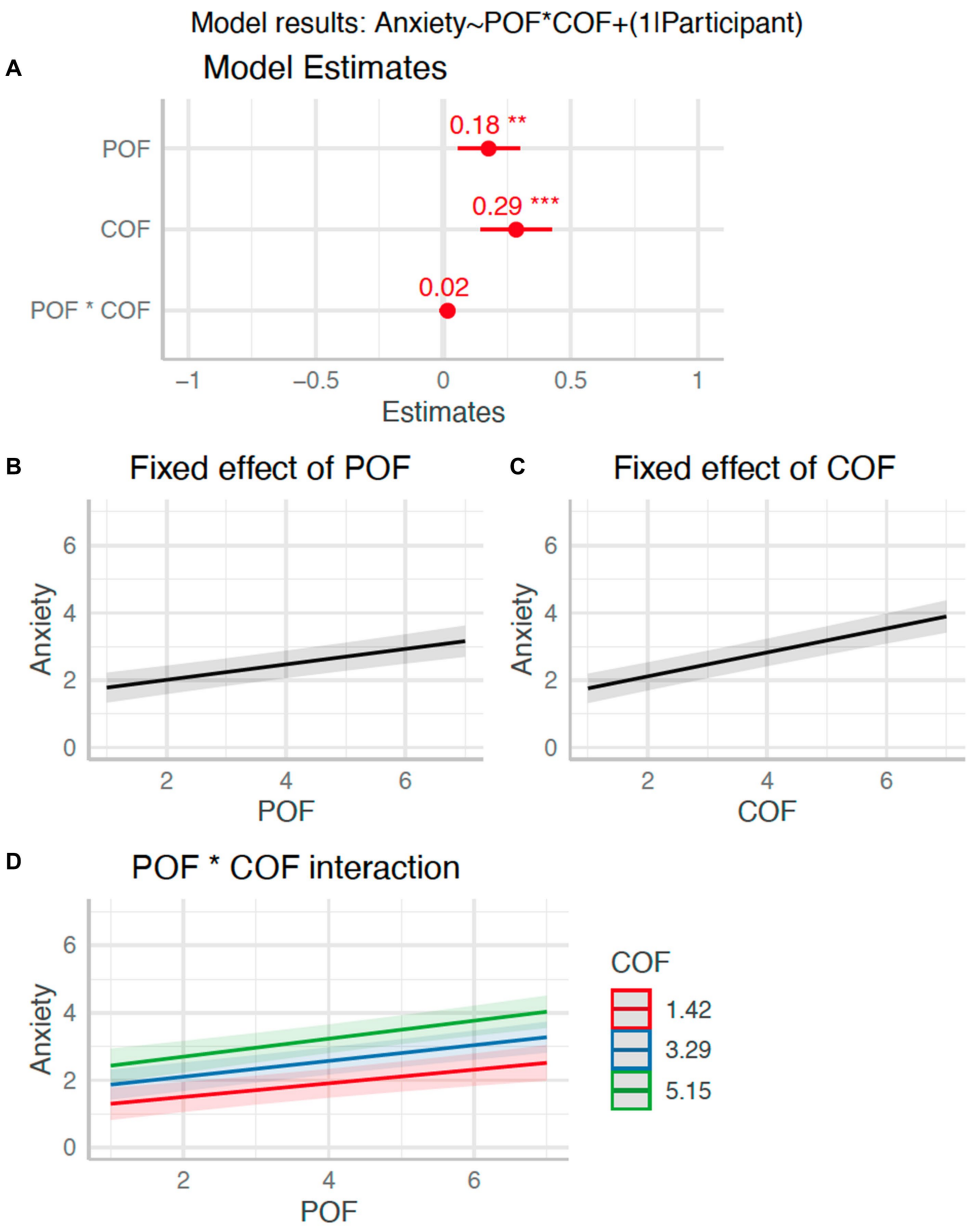
Effect of POF and COF on anxiety (H_4_). Panel **(A)**: Plot of model *β* estimates with 95%CI error bars. Significant effects are indicated by an asterisk. Panels **(B)** and **(C)**: Model estimated relationships of anxiety with POF **(B)**, and COF **(C)**. Panel **(D)**: Interaction effect illustrated for three levels of COF [mean +/− 1 standard deviation as recommended in Aiken et al. (1991)].

Next we tested whether the POF and COF ratings taken at multiple timepoints during the interception task predicted self-reported anxiety. Whilst anxiety may not have been increased by the experimental manipulations, fluctuations within conditions could still have been related to changes in POF and COF. The linear mixed model predicting anxiety with POF and COF as fixed factors and participant as a random factor had a total *R*^2^ of 0.71 and marginal *R*^2^ of 0.18 (see Figure 5). The effect of both POF (*β* = 0.18, 95%CI [0.06, 0.30], *p* = 0.004; std. beta = 0.17) and COF (*β* = 0.29, 95%CI [0.15, 0.42], *p* < 0.001; std. beta = 0.35) on anxiety were statistically significant. The interaction of POF and COF was not, however, statistically significant (*β* = 0.02, 95%CI [−0.01, 0.05], *p* = 0.251; std. beta = 0.02). This indicates that increasing ratings of probability and cost of failure predicted greater self-reported anxiety.

### 3.3. Relationship between anxiety and attention (H_5_)

Next, we tested whether momentary increases in self-reported anxiety predicted disruptions to gaze behaviours, but there was no evidence that this was the case.

#### 3.3.1. Bounce fixation pitch angle

A linear mixed model (conditional *R*^2^ = 0.60, marginal *R*^2^ < 0.01) showed that neither, anxiety (*β* = −0.08, 95%CI [−0.48, 0.33], *p* = 0.71; std. beta = −0.02), POF (*β* = 0.19, 95%CI [−0.63, 1.01], *p* = 0.65; std. beta = 0.03), COF (β = 0.07, 95%CI [−0.83, 0.97], *p* = 0.90; std. beta = 0.01), nor the POF*COF interaction (*β* = 0.01, 95%CI [−0.21, 0.19], *p* = 0.95; std. beta <0.01) significantly predicted the bounce fixation angle.

#### 3.3.2. Bounce fixation duration

**Figure 6:**
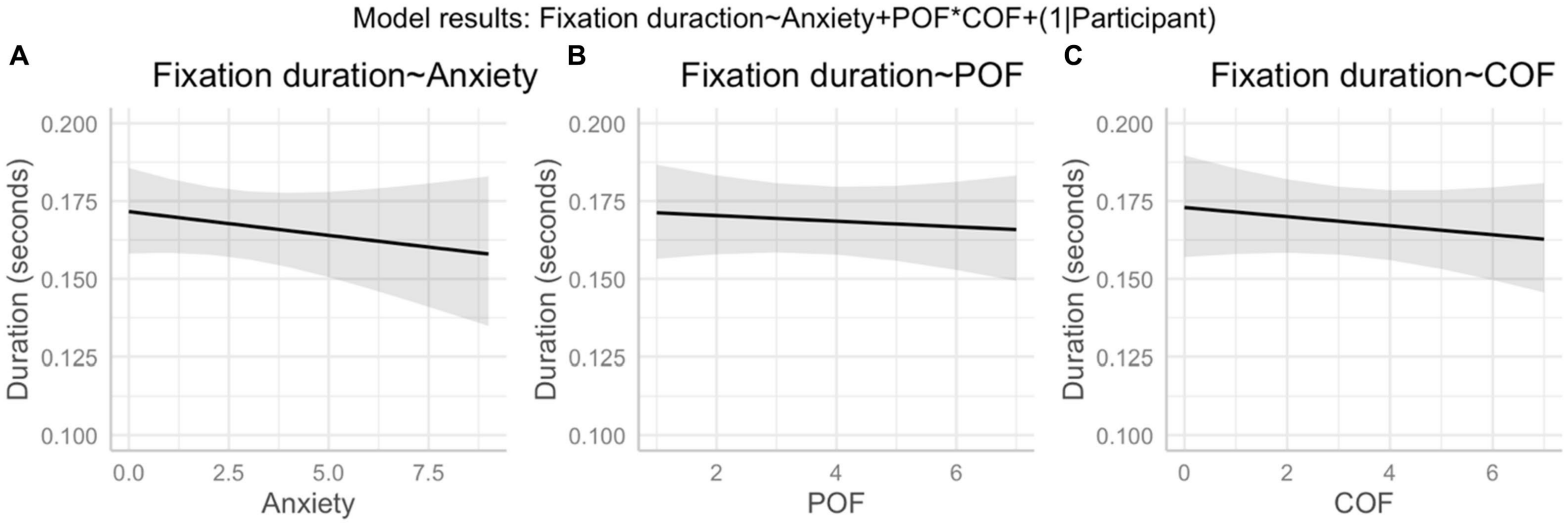
**(A–C)** Effect of anxiety on bounce fixation duration (H_5_). Shows model estimated effects with 95%CIs.

A linear mixed model (conditional *R*^2^ = 0.30, marginal *R*^2^ = 0.01) showed that neither, anxiety (*β* = −1.9*10^−3^, 95%CI [−6.0*10^−3^, 2.3*10–^3^], *p* = 0.38; std. beta = −0.02), POF (*β* = 4.7*10^−4^, 95%CI [−8.1*10^−3^, 9.0*10^−3^], *p* = 0.91; std. beta = −0.01), COF (*β* = 3.1*10^−4^, 95%CI [−9.0*10^−3^, 9.6*10^−3^], *p* = 0.95; std. beta = −0.01), nor the POF*COF interaction (β = −5.2*10^−4^, 95%CI [−2.6*10^−3^, 1.6*10^−3^], *p* = 0.63; std. beta = −0.01) significantly predicted the bounce fixation duration (see Figure 6).

### 3.4. Relationship between anxiety and performance (H_6_)

Next, we tested whether anxiety, POF, and COF were related to performance outcomes and movement variables.

#### 3.4.1. Performance

**Figure 7:**
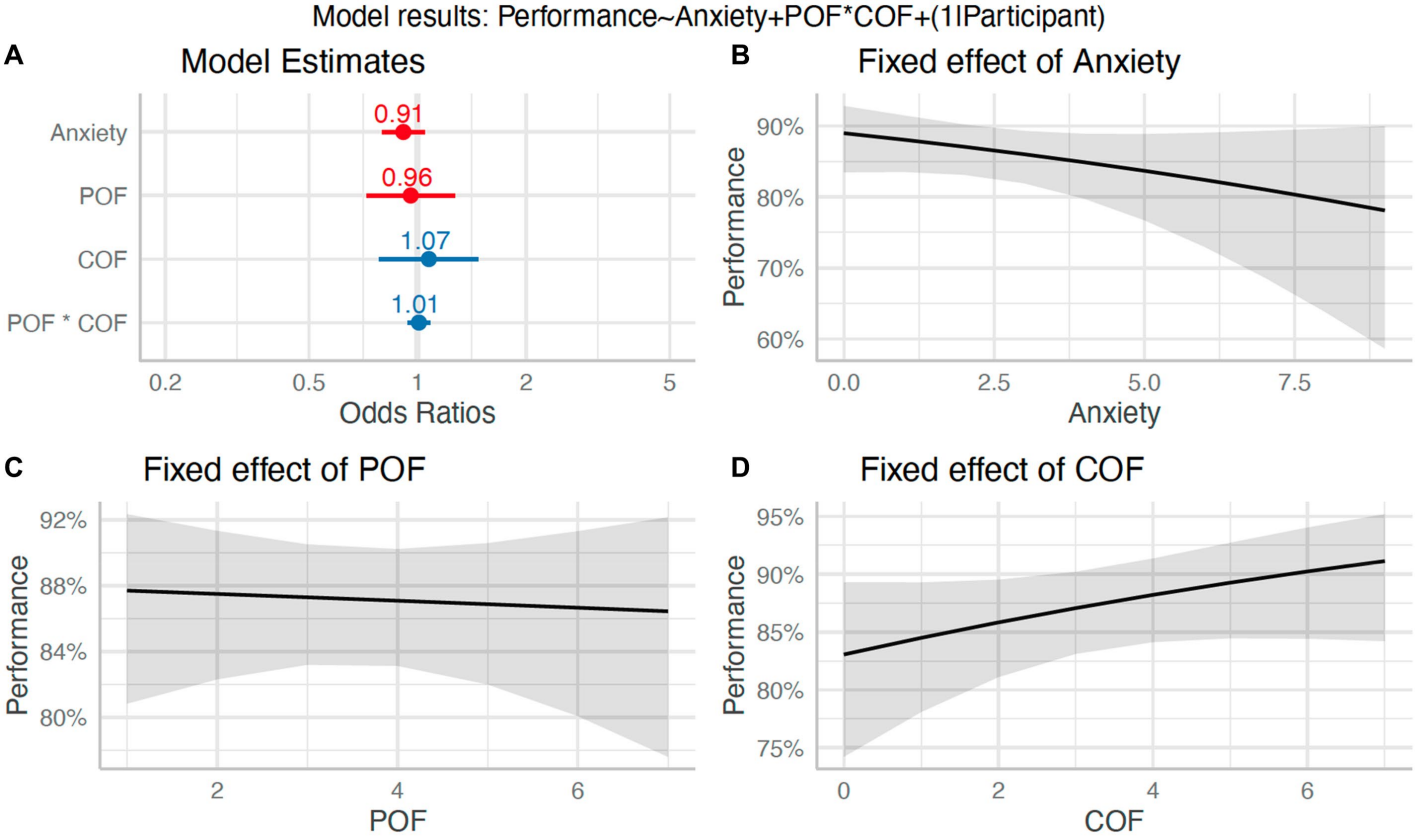
Effect of anxiety and POFCOF on performance (H_6_). Panel **(A)**: Plot of model β estimates with 95%CI error bars. Significance effects are indicated by an asterisk. Panels **B-D**: Model estimated relationships between anxiety **(B)**, POF **(C)**, and COF **(D)**, and task performance.

To address H_6_, we tested whether momentary increases in self-reported anxiety, POF, and COF (and the interaction of POF and COF) predicted disruptions to task performance. The logistic mixed model had a total *R*^2^ of 0.15 and marginal *R*^2^ of 0.01. The fixed effects of anxiety (*β* = −0.09, 95%CI [−0.23, 0.05], *p* = 0.19; std. beta = −0.17), POF (*β* = −0.04, 95%CI [−0.32, 0.24], *p* = 0.77; std. beta = −0.02), and COF (*β* = 0.07, 95%CI [−0.24, 0.39], *p* = 0.66; std. beta = 0.19) were not statistically significant. The interaction effect of COF and POF was also statistically non-significant (*β* = 0.01, 95%CI [−0.06, 0.08], *p* = 0.82; std. beta = 0.02). Therefore, there was no evidence that fluctuations in anxiety and POFCOF appraisals influenced performance on subsequent trials (see Figure 7).

#### 3.4.2. Peak swing velocity

**Figure 8:**
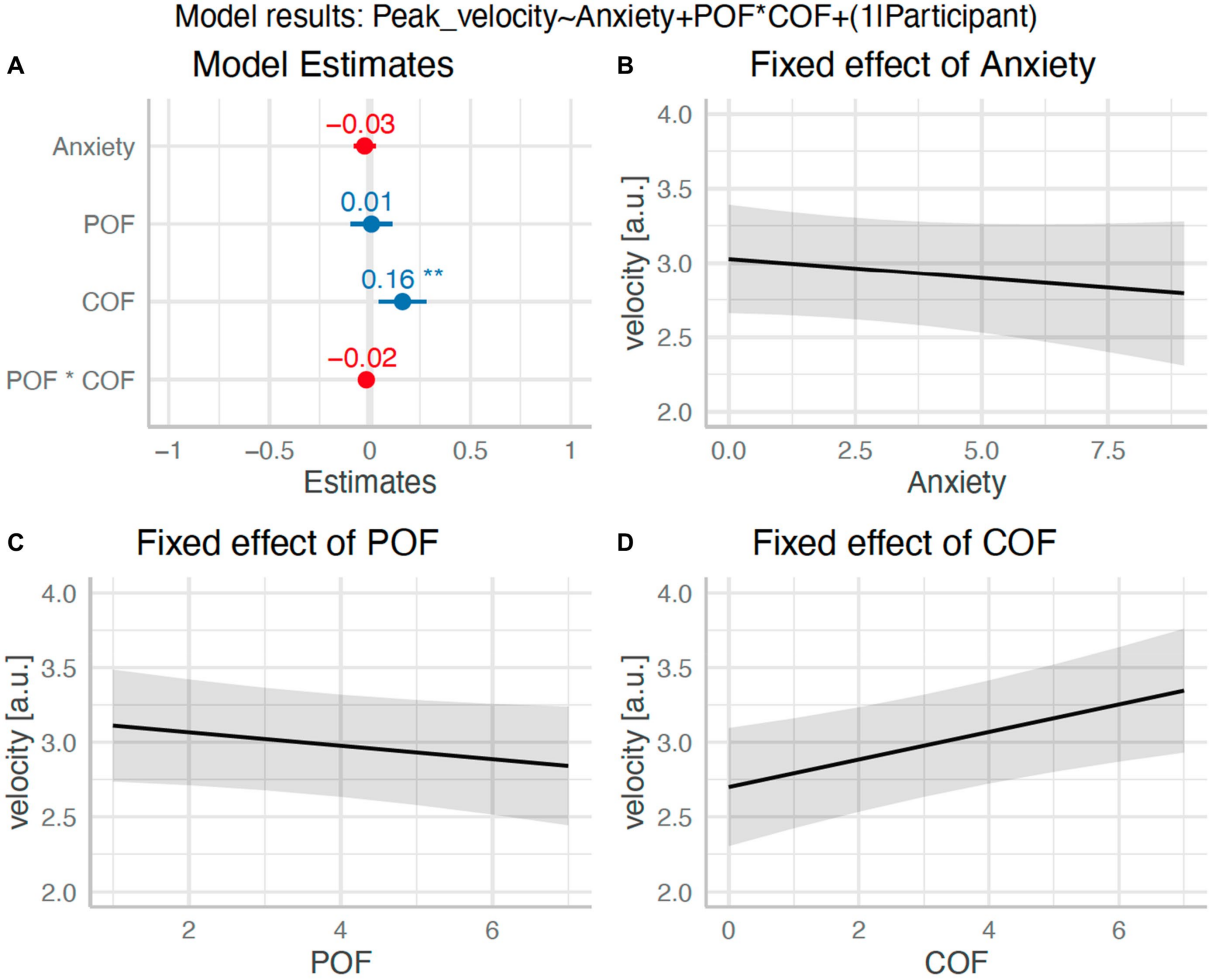
Effect of anxiety and POFCOF on swing kinematics (H_6_). Panel **(A)**: Plot of model β estimates with 95%CI error bars. Significance effects are indicated by an asterisk. Panels (**B-D)**: Model estimated relationships between anxiety **(B)**, POF **(C)**, and COF **(D)**, and swing velocity.

The linear mixed model predicting peak swing velocity with participant as a random effect had a total *R*^2^ of 0.65 marginal *R*^2^ of 0.02. The effect of anxiety was statistically non-significant (*β* = −0.03, 95%CI [−0.08, 0.03], *p* = 0.34; std. beta = −0.03), as was the effect of perceived POF (*β* = 0.01, 95%CI [−0.10, 0.11], *p* = 0.89; std. beta = −0.05). The effect of COF was, however, statistically significant (*β* = 0.16, 95%CI [0.04, 0.28], *p* = 0.007; std. beta = 0.13). There was no significant interaction of POF and COF (β = −0.02, 95%CI [−0.04, 0.01], *p* = 0.17; std. beta = −0.03). This suggests that there was a small relationship between higher COF estimates and increased swing velocity (see Figure 8).

### 3.5. Relationship between preceding errors and performance (H_7_)

**Figure 9:**
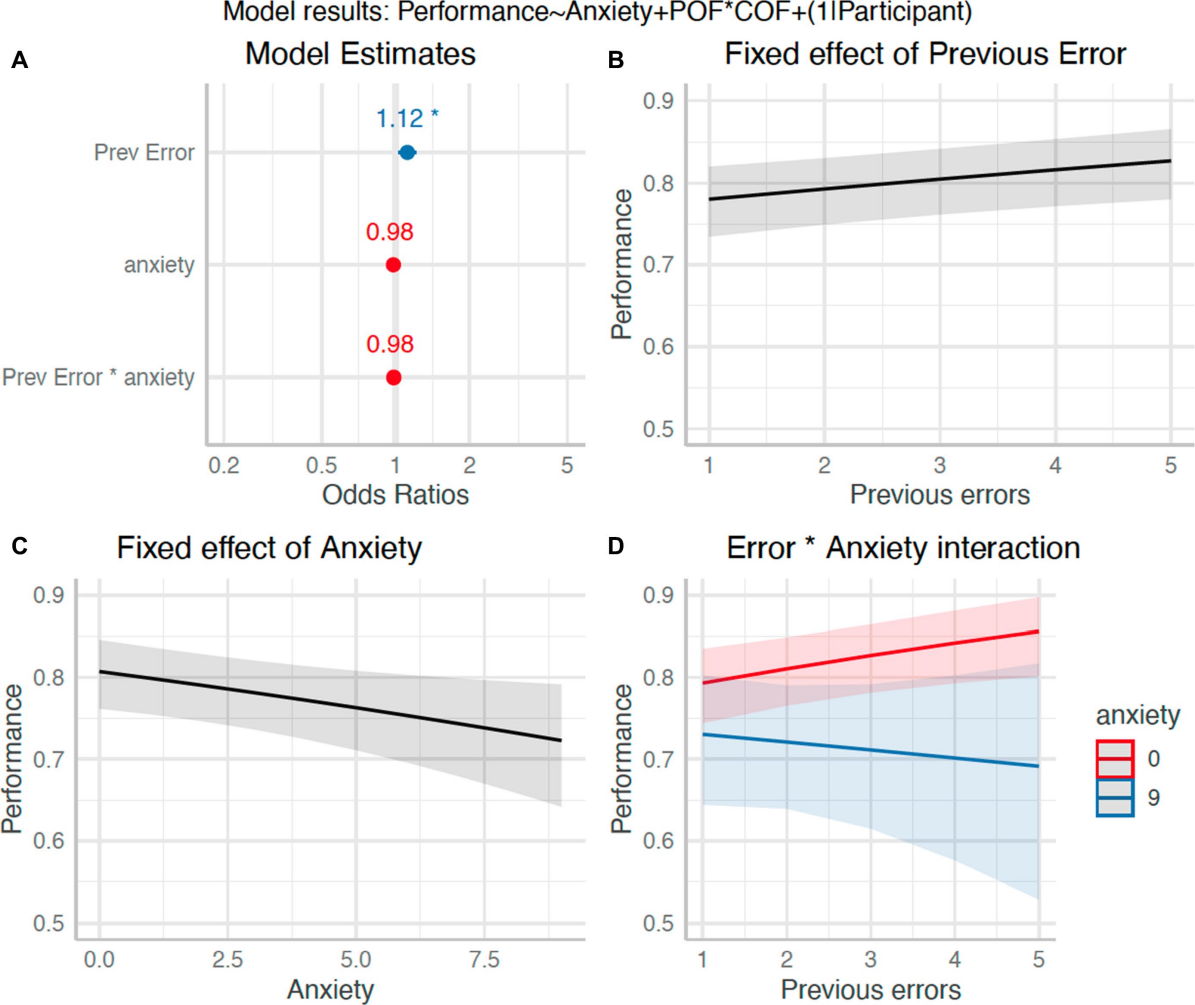
Effect of anxiety and previous errors on performance (H_7_). Panel **(A)**: Plot of model β estimates with 95%CI error bars. Significance effects are indicated by an asterisk. Panels **(B-D)**: Model estimated relationships for previous errors **(B)**, anxiety **(C)**, and their interaction **(D)**. Note a score of 1 in Panel D for previous errors actually corresponds to no previous error.

Finally, we tested whether ‘errors’ on previous trials (i.e., receiving false feedback of having made an error), predicted subsequent performance (true interception performance). Trials were coded according to the number of preceding errors (0–4), which was then used to predict performance outcome. We also entered a continuous anxiety score into the model, based on carrying forward the most recent value. A logistic mixed model with participant as random effect had a total *R*^2^ of 0.17 and marginal *R*^2^ of 0.001. The effect of a previous error was statistically significant but negligible in magnitude (*β* = 0.11, 95%CI [0.02, 0.19], *p* = 0.01; std. beta = 0.06). The effect of anxiety (*β* = −0.02, 95%CI [−0.09, 0.04], *p* = 0.53; std. beta = −0.10) and the anxiety by error interaction (*β* = −0.02, 95%CI [−0.04, 9.52e-03], *p* = 0.205; std. beta = −0.03) were not statistically significant (see Figure 9). This suggests that previous errors had limited impact performance on the subsequent trial.

## 4. Discussion

In this work we examined the pressure-performance relationship and possible precursors to anxious states, as laid out in ACTS (Eysenck and Wilson, 2016). We tested whether fluctuations in appraisals of the probability and cost of failure led to anxiety, and whether those appraisals had downstream effects on attention, movement kinematics, and performance outcomes. A more nuanced understanding of ongoing cognitive appraisals, fluctuations in anxiety, and the emotional response to previous performance errors can enable a better understanding of why movement skills break down under pressure. The results from our interceptive task largely supported the relationships between POF, COF, and anxiety as laid out in ACTS (see Figure 1). We did not, however, observe any subsequent disruptions to attention and performance.

The experimental manipulations designed to manipulate POF and COF estimates, and therefore anxiety, were only partially successful. The false feedback provided to participants was effective in increasing their perception of the likelihood of failure on subsequent trials (POF), supporting H_1_. This provides evidence in support of relationship 1 from the ACTS model in Figure 1, which predicts that previous errors will increase appraisals that future failure is more likely. The pressure conditions (induced via performance incentives and social evaluative pressure) did not have a direct impact on perceived cost of failure (COF). We did, however, observe that the interaction between pressure and failure feedback conditions led to increased COF estimates, suggesting that pressure did have an impact but only when failure was also seen as more likely (i.e., partial support for H_2_). However, neither the pressure conditions nor the failure feedback conditions had a significant effect on self-reported anxiety, leading to the rejection of H_3_. Most participants reported low anxiety scores across the experimental conditions, with relatively little between-condition variance.

To test predicted relationships 3 and 4 from the ACTS model in Figure 1 (i.e., H_4_) we assessed whether POF and COF appraisals recorded across 24 timepoints (independent of experimental condition) predicted self-reported anxiety. The strongly significant effects indicated that higher POF and COF scores did indeed predict anxiety, suggesting that these appraisals played an important role in determining anxious states. ACTS also predicts that POF and COF should have an interactive effect, such that anxiety is most likely when both are high, but we found no evidence to support this multiplicative effect. The work of Berenbaum and colleagues (Berenbaum et al., 2007; Berenbaum, 2010) has previously outlined that negative circumstances in themselves are a weak predictor of cognitive worry compared to appraisals of the probability and cost of future negative outcomes. Our findings support the idea that these intervening cognitions also play an important role in determining the onset of anxiety during visuomotor skills (see also Blascovich et al., 2004; Moore et al., 2013; Hase et al., 2019).

The failure of the experimental manipulation to induce high levels of anxiety somewhat limited our ability to test the relationship between anxiety and attention/performance (numbers 5 and 6 in Figure 1). Our pre-registered analyses did, however, focus on understanding the fluctuations in anxiety (based on the frequent anxiety thermometer measurements) rather than simply comparing blocked conditions as most previous work has done. Therefore, we were still able to assess whether small increases in anxiety led to the predicted attentional and performance disruptions. We found no evidence for a relationship between anxiety (or POFCOF estimates) and attention, movement kinematics, or performance. Many of the observed effects were, however, in the predicted directions. For instance, Figures 6, 7, 9 all show higher anxiety scores corresponded with poorer performance and disrupted attention. As the majority of anxiety scores remained in the low range, we were unable to observe the effect of higher anxiety clearly. The negative effects of anxiety on attention and performance are well established (Beilock and Carr, 2001; Hill et al., 2010; Nieuwenhuys and Oudejans, 2012; Payne et al., 2018), so there is already substantial evidence for the relationships to the right-hand side of the ACTS model (5 and 6 in Figure 1).

Finally, we tested for the presence of an error dependency effect (H_7_), where an error on a previous trial leads to a subsequent error (Harris et al., 2019). In contrast to our stated prediction, as the number of previous errors increased (i.e., participants received false feedback of more errors), there was an increase in interception rate. This effect was sufficiently small as to be inconsequential (std. beta = 0.06) but could perhaps indicate increased effort after previous failures (e.g., see Walters-Symons et al., 2017). Again, the absence of states of real anxiety may have meant that the negative impact of errors could not be observed here. For instance, Whilst no significant interaction was observed, Figure 9D suggests that with more data for higher anxiety states, there may have been a differential effect of previous errors based on whether participants were anxious or not. The confidence interval of the estimate for higher anxiety scores was very wide due to few values falling in this range. Consequently, further work is needed to investigate whether this effect, previously observed in real-world sport (Harris et al., 2019, 2021), can be detected in lab-based studies.

Previous studies using large data sets from elite sport have reported that both pressurised performance situations and previous errors are strong predictors of subsequent performance failures (Harris et al., 2019, 2021). The limitation of this previous work is that it was not possible to draw conclusions about the intervening relationships and cognitive appraisals that were responsible for the performance disruptions. The present findings go some way towards filling in this gap, suggesting that the impact of previous errors on performance may be mediated by cognitive appraisals that future failure is more likely, which then lead to greater anxiety. In addition, pressurised conditions may lead to appraisals that the cost of failure is higher, which also lead to greater anxiety. We were unable to demonstrate the subsequent impact of anxiety on attention and performance, but the large body of evidence that has tested the impact of anxiety on attention and performance means this part of the storey is already quite well evidenced.

The primary limitation of this study was the difficulty in generating higher levels of anxiety. Anxiety manipulations in lab settings can be hard to execute (e.g., see Low et al., 2022), and the failure to induce real anxiety during our task has limited the inferences we can draw. One reason the manipulation may not have worked is that each condition took 15–20 min, so even if participants felt briefly anxious it could have waned over trials. The repeated assessments of POFCOF appraisals and anxiety at multiple timepoints could also have reduced the variability in these scores, which may have been anchored to previous responses and therefore not captured true momentary experiences. Another limitation is that the attention variables may not have been as sensitive to anxiety related disruptions as they are in self-paced tasks which are more typically used (Wilson et al., 2009; Causer et al., 2011). As the effects of anxiety on attention are well-established in self-paced tasks we chose interception to try to expand the literature on interceptive skills (Causer et al., 2011; Ducrocq et al., 2016), but the variables may not have been as sensitive to any anxiety-induced disruptions. The use of bogus feedback to create the low and high failure feedback conditions could also have influenced the visuomotor variables in unexpected ways. As the outcomes reported to participants (knowledge of results) were not necessarily aligned with their knowledge of performance (e.g., visual feedback about ball and racquet movement), participants may have attempted to re-calibrate their movements based on the verbal feedback. This could have introduced additional variability in these variables that occluded effects of anxiety.

## 5. Conclusion

In the present study we sought to provide a deeper understanding of how fluctuations in situational appraisals lead to anxious emotional states, and the resultant effects on attention and performance. This work addressed a need to test the predictions of ACTS (Eysenck and Wilson, 2016) relating to the precursors to anxiety, and therefore makes a pertinent theoretical contribution to our understanding of choking under pressure. When combined with previous findings describing the effect of previous errors on performance (Harris et al., 2019, 2021) as well as the known impact of anxiety on attention and performance (Beilock and Carr, 2001; Gray, 2004; Aarts and Pourtois, 2012; Nieuwenhuys and Oudejans, 2012), the present findings contribute to a better understanding of the precursors to anxiety and the feedback loops that may maintain anxious states. In particular, our findings illustrate the importance of cognitive appraisals of the probability and cost of failure in creating anxious states. They also indicate that future work should place more focus on examining momentary fluctuations, rather than blocked pressure conditions, and should examine in more detail the cognitive appraisals that precede anxiety.

## Data availability statement

The datasets presented in this study can be found in online repositories. The names of the repository/repositories and accession number(s) can be found at: https://osf.io/vebqs.

## Ethics statement

The studies involving human participants were reviewed and approved by 22-02-02-A-02. The patients/participants provided their written informed consent to participate in this study.

## Author contributions

DH, MW, SV, FH, JL, and HR designed the study, FH, JL, and HR conducted data collection, TA and DH performed analysis, and all authors contributed to the writing of the manuscript. All authors contributed to the article and approved the submitted version.

## Funding

DH was supported by a Leverhulme Trust Early Career Fellowship.

## Conflict of interest

The authors declare that the research was conducted in the absence of any commercial or financial relationships that could be construed as a potential conflict of interest.

## Publisher’s note

All claims expressed in this article are solely those of the authors and do not necessarily represent those of their affiliated organizations, or those of the publisher, the editors and the reviewers. Any product that may be evaluated in this article, or claim that may be made by its manufacturer, is not guaranteed or endorsed by the publisher.

## Footnotes

^1^See https://developer.vive.com/resources/vive-sense/eye-and-facial-tracking-sdk/

^2^ https://osf.io/yek5s

^3^Additional analyses using post-bounce gaze tracking error, as well as swing onset times, are detailed in the supplementary files: https://osf.io/vebqs/

^4^ https://osf.io/vebqs/
